# Child implant users' imitation of happy- and sad-sounding speech

**DOI:** 10.3389/fpsyg.2013.00351

**Published:** 2013-06-21

**Authors:** David J. Wang, Sandra E. Trehub, Anna Volkova, Pascal van Lieshout

**Affiliations:** ^1^Mississauga Academy of Medicine, University of TorontoMississauga, ON, Canada; ^2^Department of Psychology, University of TorontoToronto, ON, Canada; ^3^Department of Speech-Language Pathology, University of TorontoToronto, ON, Canada

**Keywords:** prosody, emotion, production, cochlear implants, children

## Abstract

Cochlear implants have enabled many congenitally or prelingually deaf children to acquire their native language and communicate successfully on the basis of electrical rather than acoustic input. Nevertheless, degraded spectral input provided by the device reduces the ability to perceive emotion in speech. We compared the vocal imitations of 5- to 7-year-old deaf children who were highly successful bilateral implant users with those of a control sample of children who had normal hearing. First, the children imitated several happy and sad sentences produced by a child model. When adults in Experiment 1 rated the similarity of imitated to model utterances, ratings were significantly higher for the hearing children. Both hearing and deaf children produced poorer imitations of happy than sad utterances because of difficulty matching the greater pitch modulation of the happy versions. When adults in Experiment 2 rated electronically filtered versions of the utterances, which obscured the verbal content, ratings of happy and sad utterances were significantly differentiated for deaf as well as hearing children. The ratings of deaf children, however, were significantly less differentiated. Although deaf children's utterances exhibited culturally typical pitch modulation, their pitch modulation was reduced relative to that of hearing children. One practical implication is that therapeutic interventions for deaf children could expand their focus on suprasegmental aspects of speech perception and production, especially intonation patterns.

## Introduction

Modern cochlear implants (CIs) enable large numbers of prelingually deaf children to perceive speech and acquire the native language of their community by means of electrical rather than acoustic cues (Spencer et al., 1998; Svirsky et al., 2000; Blamey et al., 2001). Because the devices relay degraded pitch and spectral cues (Geurts and Wouters, 2001; Green et al., 2004), CI users have difficulty perceiving pitch sequences (Cousineau et al., 2010) such as the melodies in speech (i.e., intonation) (Hopyan-Misakyan et al., 2009; Nakata et al., 2012) and music (Vongpaisal et al., 2006; Cooper et al., 2008; Kang et al., 2009).

Intonation, perhaps the most salient aspect of speech prosody, corresponds to changes in fundamental frequency (F0) or pitch over time. Such pitch variations are often accompanied by variations in amplitude and duration (Ladd, 1996). In specific contexts, prosodic variations carry linguistic meaning, as when they distinguish nouns (e.g., *pro*ject) from verbs (e.g., pro*ject*) and statements (e.g., You're hungry) from yes/no questions (e.g., You're hungry?). Prosodic variations also provide information about a speaker's emotional state (e.g., happy, sad, angry, fearful) and intentions (e.g., approving, disapproving, sarcastic). This kind of information, pitch patterning in particular, is less accessible to listeners who use CIs. The pitch processing limitations of implants also have implications for speakers of tone languages (e.g., Mandarin, Vietnamese) where contrasting pitch height or contour can signal differences in meaning.

Research on prosody in CI users has focused mainly on perception. In general, child and adult CI users can distinguish statements (i.e., falling terminal pitch contour) from yes/no questions (i.e., rising contour) by gross periodicity cues (Rosen, 1992), but their performance is well below that of their normally hearing (NH) peers (Most and Peled, 2007; Peng et al., 2008). Their pitch processing limitations put them at an even greater disadvantage in the differentiation of vocal emotions. In one study, child CI users 7–13 years of age failed to identify utterances with neutral content that were expressed in a happy, sad, angry, and fearful manner, but they readily identified facial expressions of the same emotions (Hopyan-Misakyan et al., 2009). In other studies, child CI users identified happy and sad vocal expressions on the basis of prosodic cues alone, but they performed significantly worse than their hearing peers (Nakata et al., 2012; Volkova et al., 2013). Happy utterances typically have higher pitch and pitch variability than sad utterances (Scherer, 1986), but CI users, especially adults, may capitalize on available amplitude and duration cues (e.g., greater amplitude variation and faster speaking rate for happy utterances), as indicated by decrements in performance when those cues are unavailable (Luo et al., 2007).

Research on speech production in CI users has focused primarily on intelligibility (Peng et al., 2004a; Flipsen and Colvard, 2006), with relatively limited attention to speech prosody (but see Carter et al., 2002; Lenden and Flipsen, 2007; Peng et al., 2008) or lexical tones. The available evidence indicates that child CI users have difficulty perceiving (Barry et al., 2002) and producing (Wei et al., 2000) lexical tones. They often have difficulty producing the rising pitch contours of yes/no questions, with correlations evident between perception and production of these distinctions (Peng et al., 2008). Their differentiation of emotional expressions is predictive of their ability to imitate familiar expressions with culturally typical prosody (Nakata et al., 2012).

The ability to produce expressive variations in speech is central to communicative and social competence. Little is known, however, about child CI users' ability to produce age-appropriate distinctions in expressive prosody involving the most basic emotions such as happiness or sadness. As a first step in addressing this issue, we sought to determine the extent to which highly competent child CI users and a control sample of NH children could provide credible imitations of happy and sad prosody. In previous research, young NH children as well as child CI users produced imperfect prosodic imitations of brief Japanese utterances (Nakata et al., 2012). For NH children, the major prosodic distinctions are in place by about 5 years of age, but refinements in expressive prosody continue for some years (Cruttenden, 1985). In general, mature control of F0 is not achieved before 7 years of age (Patel and Grigos, 2006). In the present study, we recorded children's imitation of happy and sad utterances produced by a child model. Adults in Experiment 1 listened to each model utterance and imitation, rating the extent to which children's prosody matched that of the model. On the basis of NH children's advantages in the processing of F0 patterns (Vongpaisal et al., 2006; Volkova et al., 2013), we expected them to produce better imitations of the model than child CI users. Adults in Experiment 2 listened to low-pass filtered versions of the utterances that obscured the verbal content and rated each utterance on a scale ranging from very sad to very happy. We expected the happy and sad versions to be more differentiated for NH children than for child CI users. Because happy utterances embody greater prosodic variability than sad utterances (Banse and Scherer, 1996), we predicted that both NH children and child CI users would produce poorer matches of the happy utterances. Finally, with the verbal content obscured by electronic low-pass filtering in Experiment 2, we expected the utterances of child CI users to be less interpretable as happy or sad than those of NH children.

## Experiment 1

The purpose of the first experiment was to explore the ability of child CI users and NH children to imitate conventional happy and sad prosody. Previous research has indicated that child CI users can differentiate happy from sad utterances with age-appropriate stimuli and tasks (Nakata et al., 2012; Volkova et al., 2013). What remains unclear is whether they can produce distinctive happy and sad prosody. Children in the present study were required to imitate a model child's utterances, matching, as closely as possible, her expressive prosody. Adult listeners subsequently rated the closeness of each imitated utterance to the model utterance on a 10-point scale from not at all similar to extremely similar. Utterance content conflicted with prosodic form in half of the utterances. When young children are asked to judge a speaker as feeling happy or sad from utterances with conflicting verbal content and prosodic form, they typically rely on verbal content, in contrast to older children and adults who rely more on prosody (Morton and Trehub, 2001). No such judgment was required in the present experiment because children were simply asked to talk exactly like the model. Nevertheless, the conflicting content and form had the potential to interfere with children's focus on prosody and lead to poorer imitations.

### Materials and methods

#### Participants

The deaf participants, or talkers, consisted of nine bilateral CI users (five boys, four girls), 5–7 years of age (*M* = 6.0, SD = 0.7) from well-educated middle-class families who spoke English regularly at home. Of the nine CI users, six were congenitally deaf and had used their prostheses for at least 4.0 years (*M* = 4.8). Of the remaining three children, all were prelingually deaf. One became deaf in the neonatal period and two were diagnosed with progressive hearing loss at 1 year of age. Their implant experience was 3.1, 5.9, and 5.3 years, respectively. All child CI users had normal cognitive abilities. They were considered successful implant users as indicated by their speech perception skills, speech intelligibility, speech quality, and ease of communicating orally with hearing adults and peers. They had participated in auditory-verbal therapy with a focus on language acquisition for at least 2 years after implantation, and all communicated exclusively by oral means. Age of implantation, type of implant, age at recording, and etiology are shown in Table 1. The comparison sample consisted of 17 NH children (5 boys, 12 girls), 4–6 years of age (*M* = 5.2, SD = 0.8) who were also from middle-class, English-speaking families. It is common to select NH comparison groups that are slightly younger than the target CI groups to compensate for the reduced years of listening experience of child CI users (Lenden and Flipsen, 2007). Hearing was not tested in NH children, but there was no family history of hearing impairment, personal history of ear infections, or current cold, according to parents' report. The adult raters consisted of 15 NH university students (5 men, 10 women) 19–28 years of age (*M* = 23.0) who participated for partial course credit or token payment. Their hearing status was presumed to be normal by self-report.

**Table 1:** **Description of the CI sample**.

**Participant**	**Gender**	**Age at test (years)**	**Age at CI activation ear 1; 2**	**Processors (L/R when different)**	**Etiology**
CI-1	M	6.5	3.4; 3.4	Freedom	Progressive
CI-2	M	5.5	0.8; 1.7	CP810	Genetic
CI-3	M	5.6	1.1; 1.1	Freedom	Genetic
CI-4	F	6.9	1.0; 3.6	SPrint/Freedom	Genetic
CI-5	F	7.2	2.5; 4.0	Freedom	Progressive
CI-7	M	5.3	0.9; 1.8	Freedom	Genetic
CI-8	M	6.1	0.8; 1.5	Freedom	Genetic
CI-12	F	6.3	1.0; 3.5	SPrint/Freedom	Unknown
CI-17	F	5.1	1.1; 3.3	Freedom	Genetic

#### Apparatus

Children's utterances were recorded in a double-walled, sound-attenuating booth (Industrial Acoustics Company) with a microphone (Sony F-V30T) and external sound card (SoundBlaster X-Fi Fatal1ty) linked to a computer workstation outside the booth running Windows XP and Audio Recording Wizard version 4 (NowSmart) software. Audio stimuli for imitation were presented via an amplifier (Harman/Kardon HK3380) outside the booth and two loudspeakers (Electro-Medical Instrument Co.), one on either side of the seated child at a distance of 80 cm and 45° azimuth. NH undergraduates were tested in the same sound-attenuating booth with audio stimuli presented over the loudspeakers. Rating tasks were presented through an interactive computer program that automatically recorded response selections on a 17-inch touch-screen monitor (Elo LCD TouchSystems).

#### Stimuli

A 10-year-old native speaker of English (female) produced several versions of sentences (see Table 2) in a happy and sad manner. The most clearly articulated and prosodically natural versions were selected, by consensus, as model utterances. High-quality digital sound files (44.1 kHz, 16-bit, monaural) were created by means of a digital audio editor (Sound Forge 6.0). Child CI users and NH children began by playing an interactive game in which they copied whatever the experimenter said, doing so exactly the way she said it. After this orientation phase, they were instructed to listen to each recorded utterance of the girl (the model), attempting to imitate it as closely as possible. They were told to pay particular attention to the way the girl spoke, copying her happy or sad way of talking. Then the model utterances were presented, one by one, at approximately 65 dB SPL, and children were recorded while imitating each utterance. The child model presented each utterance in both a happy and sad manner for a total of 16 utterances. Children's imitations were normalized for root-mean-square amplitude by means of PRAAT speech analysis and synthesis software (v. 5.3.17; Boersma and Weenink, 2008). Stimuli were played to the adult raters at approximately 65 dB SPL. Only four of the eight utterances (1, 4, 6, 7 from Table 2, selected randomly) were used in the rating task because of time constraints of testing (1 h session). The final stimulus set for adult listeners consisted of 8 utterances (four happy four sad versions) from 26 children for a total of 203 utterances (5 of the potential set of 208 utterances were missing because of instances in which children failed to provide an imitation). Sample happy and sad utterances from the child model and from a child CI user are provided in Supplementary Materials.

**Table 2:** **Sentences imitated by children**.

1. Look. My bike is broken
2. I lost my new red crayon
3. The doggie ate my birthday cake
4. My friend can't come to play
5. I had so much fun at the park
6. My dad gave me a present
7. Look at that cute puppy
8. Wow. What a pretty rainbow

#### Procedure

Normally hearing undergraduates were tested individually. Eight utterances from each child were presented, half happy and half sad versions. Participants listened to each model utterance followed by the imitation of each child CI user and NH child in random order and rated how closely each utterance matched the model on a 10-point scale (1 = not similar at all to 10 = extremely similar). Prior to the actual test trials, participants completed a practice phase with utterances that were not included in the test phase. Participants were instructed to base their ratings on utterance intonation rather than content. In other words, they were encouraged to ignore the occasional word errors that children made. They were not told anything about children's age or hearing status.

PRAAT software was used to extract the acoustic features in children's imitations. Vowel boundaries were demarcated to include the entire vowel from spectrographic depictions of the model utterances and imitations, after which estimates of F0 (mean, SD, range), duration, and intensity variability (SD) were obtained automatically by means of a custom-made script.

### Results

**Figure 1:**
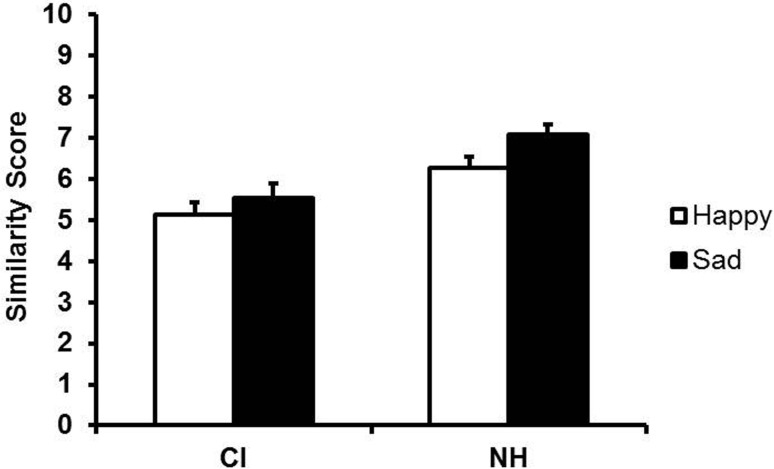
**Mean ratings of child CI users' and NH children's imitations of the model's happy and sad utterances (content intact)**. Error bars indicate standard errors.

An analysis of variance (ANOVA) with hearing status (CI, NH) as a between-subjects factor and content/form (consistent, conflicting) as a within-subjects factor, revealed a significant effect of hearing status, *F*(1, 29) = 172, *p* < 0.001, reflecting better performance of NH children, and a significant effect of content/form, *F*(1, 29) = 26.47, *p* < 0.001, but no interaction between hearing status and content/form. Unexpectedly, children matched the model better for conflicting than for consistent utterances. Examination of the model's consistent and conflicting utterances indicated systematically lower F0 (i.e., slightly less happy-sounding) for inconsistent happy than for consistent happy utterances. Because the conflicting utterances did not put children at a disadvantage and had comparable effects for both groups, the consistent and conflicting utterances were combined in subsequent analyses. Adults' mean ratings of the imitations of happy and sad utterances by child CI users and NH children are shown in Figure 1. An ANOVA with hearing status (CI, NH) as a between-subjects factor and utterance type (happy or sad) as a within-subjects factor revealed a main effect of hearing status, *F*(1, 14) = 148, *p* < 0.001. This effect reflected lower ratings for child CI users' imitations (*M* = 5.33, SD = 0.32) than for those of NH children (*M* = 6.68, SD = 0.24). In fact, child CI users received significantly lower ratings than NH children on happy utterances (CI: *M* = 5.13, SD = 1.22; NH: *M* = 6.28, SD = 1.03), *t*(14) = 12.98, *p* < 0.001, as well as sad utterances (CI: *M* = 5.53, SD = 1.36; NH: *M* = 7.07, SD = 0.98), *t*(14) = 8.81, *p* < 0.001). There was also a main effect of utterance type, *F*(1, 14) = 10.01, *p* = 0.007, reflecting higher overall ratings for sad utterances (*M* = 6.30, SD = 0.29) than for happy utterances (*M* = 5.71, SD = 0.29). Finally, there was a significant interaction between hearing status and utterance type, *F*(1, 14) = 5.48, *p* = 0.035, which arose from greater rating differences between the happy and sad utterances of NH children than child CI users. In fact, the rating differences for NH children's happy and sad utterances were highly significant, *t*(14) = −3.81, *p* = 0.002, and the same trend was evident for child CI, *t*(14) = −1.96, *p* = 0.07.

**Figure 2:**
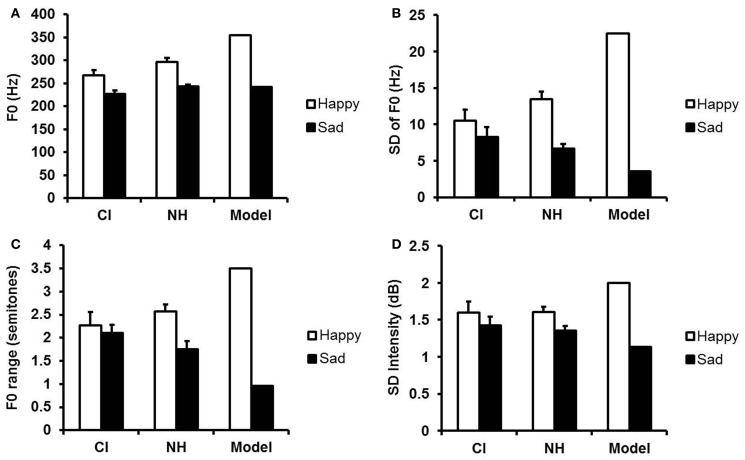
**Acoustic features of the happy and sad utterances of child CI users, NH children, and the model. (A)** Mean F0 (Hz); **(B)** mean variability (SD) of F0 (Hz); **(C)** mean F0 range (semitones); **(D)** mean variability (SD) of intensity (dB). Error bars indicate standard errors.

Acoustic features of the child model's utterances and children's imitations are shown in Figure 2. It is apparent that the child model's happy and sad utterances were much more distinct in pitch level, pitch variability, pitch range, and intensity variability than were those of the young child imitators, whether hearing or deaf. Nevertheless, the happy and sad imitations of both groups of children were still distinct. The model's happy and sad utterances differed most from the imitators in their greater variability in F0 and F0 range. Likewise, the NH children differed most from child CI users in these indices of variability.

## Experiment 2

Experiment 1 revealed that NH children imitated the model's happy and sad utterances more effectively than child CI users did, but both groups produced clearly differentiated utterances. In addition, both groups produced better imitations of sad than happy utterances. Although the acoustic analyses revealed distinctive cues for happy and sad utterances, the model's cues were considerably more distinctive than those of the imitators. The question of interest here was whether the imitators' utterances would be interpretable as happy and sad on the basis of prosodic cues alone, that is, when listeners had no access to verbal content.

### Materials and methods

#### Participants

The participants were 16 NH undergraduates (4 men, 12 women) 19–28 years of age (*M* = 21.1), who received partial course credit or token payment for their participation. Their hearing status was presumed to be normal by self-report. An additional participant was tested but excluded from the final sample for failure to provide ratings for several utterances.

#### Apparatus

The apparatus was the same as in Experiment 1.

#### Stimuli

A randomly selected subset of the happy and sad imitations of child CI users and NH children from Experiment 1 – utterances 1, 2, 4, 5, 6, and 7 in Table 2 – was normalized for root-mean-square amplitude and low-pass filtered with a cutoff frequency of 500 Hz (via PRAAT). Low-pass filtering preserved frequencies below 500 Hz and attenuated higher frequencies, which made the verbal content unintelligible while retaining cues to emotion such as intonation, speech rate, and speech rhythm (Ben-David et al., 2013). The stimuli were presented at approximately 65 dB SPL. The low-pass filtered versions of a happy and sad utterance from one CI user can be found in Supplementary Materials.

#### Procedure

Participants were tested individually. Happy and sad versions of each utterance (total of 12 utterances per child) were presented for a total of 304 utterances (26 children × 12 utterances each = 312 minus the occasional missing imitations). Participants listened to each filtered utterance and rated how happy or sad each sounded on a 7-point scale (1 = very sad, 4 = neutral, 7 = very happy). Unlike the rating scale in Experiment 1, which involved a single dimension of similarity, the present bipolar scale had a neutral midpoint (neither sad nor happy). Testing was preceded by a familiarization phase to provide exposure to the sound quality of filtered utterances and practice rating the utterances on the happy/sad scale. Utterances in the familiarization phase differed from those in the test phase.

### Results

**Figure 3:**
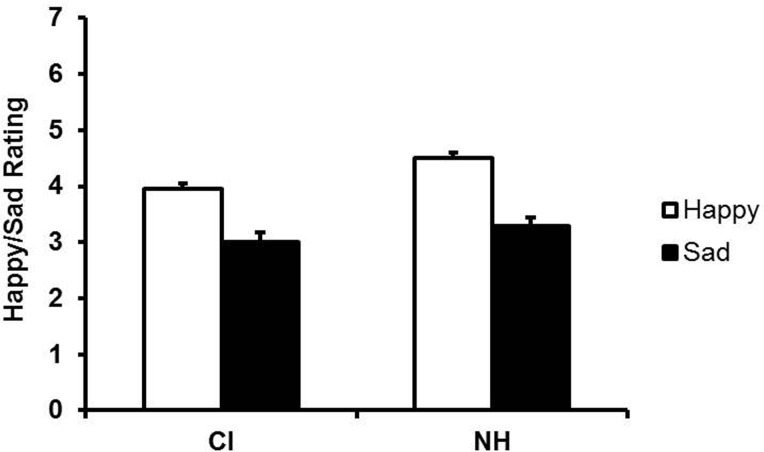
**Mean ratings of the low-pass filtered imitations (i.e., content unintelligible) of child CI users and NH children as happy- or sad-sounding**. Error bars indicate standard errors.

Mean ratings for happy and sad utterances produced by child CI users and NH children are shown in Figure 3. Note that the mean rating for NH children appears to be above the neutral midpoint of four (i.e., in the happy zone) for happy utterances but slightly below the midpoint for child CI users. Note also that both groups achieved mean ratings below four (i.e., in the sad zone) for sad utterances. This clustering of ratings close to the neutral midpoint suggests that, on average, the filtered versions did not sound particularly happy or sad. To ascertain whether adults provided differential ratings of the happy and sad utterances, we examined differences in mean ratings (happy minus sad ratings) for all happy and sad utterances of both groups. One sample *t*-tests indicated that the difference scores significantly exceeded zero for child CI users (*M* = 0.95, SD = 0.68), *t*(15) = 5.60, *p* < 0.001, as well as NH children (*M* = 1.20, SD = 0.74), *t*(15) = 6.48, *p* < 0.001. A paired samples *t*-test revealed that the difference scores were significantly larger for NH children *t*(15) = 3.77, *p* = 0.002, than for child CI users, reflecting adults' greater ease of identifying the emotional intentions of NH children from prosodic cues alone.

**Figure 4:**
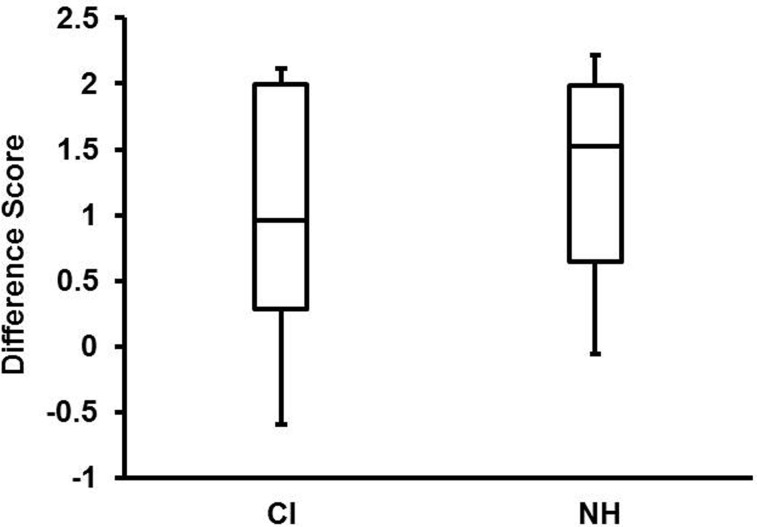
**Box plots of difference scores (happy/sad ratings of happy utterances minus ratings of sad utterances) for child CI users and NH children**. Top and bottom lines of each box indicate the 75th and 25th percentile, respectively. Lines within each box indicate the median. Top and bottom of the whiskers indicate the highest and lowest scores.

As can be seen from the boxplots in Figure 4, there were large individual differences in the efficacy of child CI users' prosodic cues. Although the emotional intentions of NH children were more transparent than those of child CI users, difference scores for the top quartile of child CI users (2.00) and NH children (1.98) were roughly equivalent. Because pitch level and pitch variability are particularly distinctive markers of happy vocal affect (Scherer, 1986), the mean F0 and SD of F0 were compared for the happy and sad utterances of NH children and child CI users by means of paired-sample *t*-tests (with Bonferroni corrections for multiple tests). Happy utterances of NH children had significantly higher mean F0, *t*(17) = 5.92, *p* < 0.001, and SD of F0, *t*(17) = 5.04, *p* < 0.001, than sad utterances. Mean F0 also differentiated the happy and sad utterances of child CI users, *t*(8) = 3.86, *p* = 0.049, but F0 variability did not. Again, there were large individual differences in child CI users' use of F0 and F0 variability to distinguish their happy from their sad utterances. Despite the modest sample size of child CI users (*n* = 9), mean F0 difference of happy and sad utterances was highly correlated with adults' difference scores (ratings), *r*(7) = 0.71, *p* = 0.03. The correlation between F0 variability and adult difference scores did not reach conventional significance levels, *r*(7) = 0.6, *p* = 0.086.

## Discussion

The goal of the present study was to ascertain the ability of child CI users and young NH children to signal happiness and sadness by speech prosody alone. Children 4–7 years of age imitated utterances with conventional happy and sad prosody that had been produced by a 10-year-old child. Half of the model utterances had happy content and half had sad content, but all utterances were produced in both a happy and a sad manner. Adults listened to the model's version of an utterance before hearing each child's imitation of that utterance, rating how closely the imitation matched the model. In principle, the divergent content and expressive style could have been a source of confusion, leading to less adequate imitations of those utterances than for utterances with consistent content and style. Surprisingly, children, both hearing and deaf, produced better prosodic matches in the context of inconsistent content and prosody, which indicates that they can focus on prosody when imitating utterances even though they have difficulty doing so in emotional judgment tasks (Morton and Trehub, 2001). The lower mean F0 of the model's inconsistent happy utterances, like their sad utterances, may have contributed to children's greater ease of imitation. It is also possible that the discordant messages captured children's attention, increasing their sensitivity to the acoustic cues and leading to better imitations.

Both groups of children produced better imitations of sad prosody than happy prosody. Unquestionably, happy prosody is more engaging than sad prosody for listeners in general and children in particular, but it is more difficult to reproduce because of its greater pitch range and modulation (Banse and Scherer, 1996). For example, young children's imitations of expressive utterances such as exclamations or simulated animal sounds (e.g., meow) reveal a considerably smaller pitch range than that of older children (Nakata et al., 2012).

Although NH children and child CI users showed similar overall patterns of performance, their levels of performance differed significantly. NH children produced better imitations of happy and sad messages than did the child CI users, as reflected in adults' ratings. The imitations of child CI users, on average, were modest in quality rather than being poor, with mean ratings near the midpoint on the 10-point scale of similarity to the model. Acoustic analyses revealed that both groups of children used distinctive F0 cues for their happy and sad utterances, but even NH children, on average, failed to produce happy and sad utterances that were as highly contrastive in mean F0 and F0 variability as those of the older child model (see Figure 2). What is impressive, however, is that the best child CI users were equivalent to the best performing NH children. Sample utterances from the model and from one high-performing CI user can be found in Supplementary Materials.

The modest pitch modulation in many children's utterances increased the difficulty of judging their low-pass filtered utterances as happy or sad, as evident in the ratings and in listeners' comments after completing the task. Amplitude normalization removed obvious cues such as the higher overall amplitudes of happy than sad utterances although it preserved the greater amplitude variability of happy utterances. In general, speaking rate, especially vowel duration, distinguishes adults' happy from sad utterances (Scherer, 1986), but even the model did not use timing cues for such purposes. Although adults did not rate the utterances as particularly happy or sad, they assigned significantly higher (happier) ratings to the happy versions than to the sad versions both for NH children and for child CI users. Our finding of more differentiated ratings for NH children's utterances than for those of child CI users is consistent with reports of lesser prosodic expressiveness by child CI users (Lenden and Flipsen, 2007; Nakata et al., 2012). It is important to note, however, that distinctive productions of happy and sad speech remained distinctive after low-pass filtering (see Supplementary Materials for examples).

The happy and sad utterances of NH children differed in mean F0 and F0 variability, but F0 variability did not distinguish the happy and sad utterances of CI users. Perhaps the cluster of acoustic cues that predicts listeners' ratings is different for NH children and child CI users. Given the emotion perception (e.g., Hopyan-Misakyan et al., 2009) and prosodic production limitations (Lenden and Flipsen, 2007) reported in previous studies, child CI users' performance in the present study is impressive. The use of imitations rather than spontaneous speech reduced the processing demands on child CI users, perhaps optimizing performance. For example, emphatic stress is less problematic in imitated (Carter et al., 2002) than in spontaneous (Lenden and Flipsen, 2007) speech.

Unquestionably, device limitations increase the cognitive effort of listening in general (Pals et al., 2012) as well as emotion perception and production difficulties in particular (Peng et al., 2008; Hopyan-Misakyan et al., 2009; Nakata et al., 2012; Volkova et al., 2013). Remarkably, however, they do not preclude successful performance on such tasks by the best CI users (Peng et al., 2004b; Nakata et al., 2012; Volkova et al., 2013), including the top performers in the present study. The highest performing child CI users had a number of background factors associated with favorable outcomes, including early implantation (Tomblin et al., 2005) and highly educated and motivated parents (Teagle and Eskridge, 2010). Interestingly, these “star” children were also taking music lessons, which may have helped focus their attention on the pitch patterns and rhythms of speech. There is evidence linking music lessons in childhood to improved pitch perception (Chen et al., 2010) and enhanced linguistic abilities (Moreno et al., 2009). One practical implication of the findings is that therapeutic interventions for child CI users, which focus primarily on speech perception and speech intelligibility and secondarily on some aspects of prosody would do well to expand their focus on emotional expressiveness.

In short, young child CI users effectively reproduce the prosody of happy and sad utterances, but their reproductions are less accurate than those of NH children. Despite the fact that child CI users provide fewer cues than NH peers to signal their happy and sad intentions, adults interpret their intentions at better than chance levels on the basis of prosodic cues alone. Child CI users, who were 5–7 years of age, spent one or more years without functional hearing, so their chronological age does not reflect their cumulative listening experience, as it does for NH children. It is important to ascertain whether the gap between the prosodic skills of young child CI users and NH children narrows or disappears over time either spontaneously or as a result of intervention.

## Author note

All research reported in this paper was approved by local ethical committees. Funding for this project was provided by grants from the Social Sciences and Humanities Research Council of Canada (Sandra E. Trehub) and from the Comprehensive Research Experience for Medical Students at the University of Toronto (David J. Wang). We thank the families of participating children for their cooperation and Judy Plantinga for her assistance in implementing the experiments.

### Conflict of interest statement

The authors declare that the research was conducted in the absence of any commercial or financial relationships that could be construed as a potential conflict of interest.
